# Peptide Designs for Use in Caries Management: A Systematic Review

**DOI:** 10.3390/ijms24044247

**Published:** 2023-02-20

**Authors:** Olivia Lili Zhang, John Yun Niu, Ollie Yiru Yu, May Lei Mei, Nicholas Stephen Jakubovics, Chun Hung Chu

**Affiliations:** 1 Faculty of Dentistry, The University of Hong Kong, Hong Kong, China; 2 Faculty of Dentistry, The University of Otago, Dunedin 9054, New Zealand; 3 School of Dental Sciences, Newcastle University, Newcastle upon Tyne NE2 4BW, UK

**Keywords:** antimicrobial, caries, peptides, prevention, remineralisation

## Abstract

The objective of this study was to review the design methods that have been used to create peptides for use in caries management. Two independent researchers systematically reviewed many in vitro studies in which peptides were designed for use in caries management. They assessed the risk of bias in the included studies. This review identified 3592 publications, of which 62 were selected. Forty-seven studies reported 57 antimicrobial peptides. Among them, 31 studies (66%, 31/47) used the template-based design method; 9 studies (19%, 9/47) used the conjugation method; and 7 studies (15%, 7/47) used other methods, such as the synthetic combinatorial technology method, the de novo design method and cyclisation. Ten studies reported mineralising peptides. Seven of these (70%, 7/10) used the template-based design method, two (20%, 2/10) used the de novo design method, and one study (10%, 1/10) used the conjugation method. In addition, five studies developed their own peptides with antimicrobial and mineralising properties. These studies used the conjugation method. Our assessment for the risk of bias in the 62 reviewed studies showed that 44 publications (71%, 44/62) had a medium risk and that 3 publications had a low risk (5%, 3/62). The two most common methods for developing peptides for use in caries management that were used in these studies were the template-based design method and the conjugation method.

## 1. Introduction

Dental caries is the most prevalent disease suffered by humans worldwide. The distribution of this disease has an unequal burden, as it has a particularly high impact on those from lower socioeconomic groups [1]. For example, in the United States, there are clear disparities in the caries burden across the population, which reflects unequal access to oral health care [2]. Caries result from a perturbation in the balance between tooth minerals and microbial biofilms [3], which are microbial communities that adhere to and cover hard tooth tissues [4]. The extracellular polymeric matrix produced by bacteria and the host can improve the biofilm community’s ability to survive in different environments [4]. Carious lesions form in the oral cavity due to the acid production of cariogenic biofilms [5]. In many cases, an increase in the abundance of *Streptococcus mutans* is associated with the development of dental caries [6]. *S. mutans* is highly acidogenic and aciduric, which is likely to contribute to its ability to promote enamel demineralisation [7]. Acidogenic fungi, such as *Candida albicans,* also contribute to the progression of caries [8].

Effective antibiofilm strategies to control the growth of cariogenic bacteria, such as *S. mutans*, are essential for caries management [9]. However, traditional antibiotic treatment is unhelpful for controlling caries because extracellular polymeric matrix substances can protect the resident microbes in the biofilm [10]. In addition, high concentrations of antibiotics further promote resistance mutations in microorganisms [10]. Moreover, antibiotics could change the health of the oral microbial environment, which could lead to opportunistic infections [11]. Hence, a safe and effective treatment is necessary to overcome the failure of antibiotic therapy in caries management.

The traditional treatment for caries is to drill the damaged tissue and fill it with restoration biomaterials [12]. However, this strategy could result in the loss of a healthy tooth structure, which could lead to another cycle of drilling and filling. As a result, the small filling will eventually be replaced by a large filling, and more healthy teeth will be removed. Over the long term, this cycle will increase individual burden and public health costs. To overcome these limitations and further extend the permanence of natural teeth, the caries management philosophy should change to minimal intervention treatment for early caries [13]. Thus, researchers have developed novel bioactive materials for caries management that have antibacterial and mineralising properties [14], including peptide-based materials.

Among these peptide-based materials are antimicrobial peptides, which can be used against broad-spectrum microorganisms. These present a promising strategy for caries management [11,15]. There is an increasing amount of in vitro evidence indicating that numerous antimicrobial peptides have significant efficacy in inhibiting bacteria [16]. Antimicrobial peptides rarely produce resistance because they usually attack multiple hydrophobic and polyanionic bacterial targets [17], which, presumably, overcome microbial resistance [18]. All organisms, including bacteria, fungi, plants and animals, can produce antimicrobial peptides [19]. For example, nisin is one of the earliest reported antimicrobial peptides and is produced by the Gram-positive bacterium *Lactococcus lactis* [20]. Although it has been used for 50 years as a food preservative, no significant microbial resistance has been observed [19].

Most antimicrobial peptides consist of between 10 and 50 amino acids with a positive charge and approximately 50% hydrophobic residues [21]. Antimicrobial peptides can exist with different secondary structures. Typical structures include α-helical, β-sheet, cyclisation and linear extensions [22]. The most common structure is α-helical, because abundant natural α-helical antimicrobial peptides have been isolated [23]. The hydrophilic and hydrophobic residues of α-helical antimicrobial peptides make them amphipathic when interacting with targeted membranes [24]. The β-sheet structure contains at least two β-strands, adopting a β-hairpin-like conformation [25]. The β-hairpin-like structure is critical for the antimicrobial activities of β-sheet antimicrobial peptides [26]. Another group of antimicrobial peptides lack α-helical and β-sheet domains and are sometimes known as extended or loop peptides [21]. Some peptides, such as human β-defensin-1 (HBD-1), contain both α-helical and β-sheet domains, which could strongly target membranes [27]. Positively charged antimicrobial peptides can initially bind to the negatively charged bacterial membrane. The antimicrobial peptide’s hydrophobic tail can be inserted into the bacterial lipid bilayer. This causes bacterial membrane damage and cytoplasmic leakage, which results in the death of bacterial cells [21,28].

Antimicrobial peptides are effective against a wide spectrum of microbes in the oral cavity, including bacteria, fungi and viruses [29]. These mechanisms include both the direct killing of pathogens and interactions with the host’s immune response [29]. The major antimicrobial peptides in the oral cavity are cathelicidins, defensins and histatins. Natural cathelicidins have different structures [30]. LL-37 is the only cathelicidin in human beings and has an α-helical structure. It acts against *S. mutans* and *C. albicans* by interacting and causing pore formation in the cell walls [21]. Another common natural group of antimicrobial peptide is defensins, which can be divided into two subfamilies: α-defensin (HNP) and β-defensin (hBD) [29]. Studies have indicated that defensins and histatin-5 are effective against *S. mutans* [11]. In addition, hBD-2 and hBD-3 are effective against *C. albicans* [29]. However, natural antimicrobial peptides are unstable in the oral environment [22].

Researchers are working to develop novel synthetic antimicrobial peptides with improved stability and antimicrobial activities to aid in caries management [15]. For example, they designed KR12-KAKE by mimicking LL-37, which has shown activity against *S. mutans* [31]. In addition, some synthetic antimicrobial peptides have been developed through physiochemical modifications such as fusing functional sequences to retain or enhance their activity in various environments [32]. For example, the novel antimicrobial peptide C16G2 can target *S. mutans* through the fusion of the following three parts: CSP_C16_ —a *S. mutans* targeted domain; GGG—a linker; and G2—a broad-spectrum antimicrobial peptide [33]. To manage caries, promoting tooth remineralisation is as essential as inhibiting biofilms [11]. Thus, developing mineralising peptides to induce biomimetic remineralisation is also a strategy for caries management [34]. Enamel matrix proteins, such as amelogenin, can inhibit demineralisation and promote remineralisation [35]. In addition to developing novel antimicrobial peptides, researchers have also developed mineralising peptides, using different methods [36]. For example, QP5, which is derived from amelogenin, has been shown to promote the remineralising of enamel caries [35]. In addition, a de novo designed, self-assembling peptide—such as P_11-4_—can construct a three-dimensional matrix structure for enamel regeneration [34]. Researchers are also developing dual-functional peptides for use in caries management, which have antimicrobial and mineralising properties [37]. Niu et al. created GA-KR12 by grafting gallic acid—as the mineralising functional domain—to the antimicrobial peptide KR12, which is derived from LL-37 [38]. In vitro studies have demonstrated that GA-KR12 inhibited the growth of *S. mutans* biofilm and induced the remineralisation of dentin and enamel caries lesions [17,37].

Researchers are interested in developing peptides for caries management [15]. Most of the research are laboratory studies and only a few studies are pre-clinical trials [39]. Although almost all the laboratory studies that we reviewed showed that peptides are effective in caries management, the clinical evidence is not strong, which is significant as high-quality basic research is the foundation of translational research [40,41]. Many literature reviews that have focused on the type and function of peptides have been published [11]. However, no review has comprehensively or systematically identified the range of novel peptide designs that have been developed to manage caries and the different design methods that have been employed. Therefore, this systematic review aimed to comprehensively investigate the design methods used in the creation of peptides for caries management and to assess the quality of these studies.

## 2. Results

A total of 3592 publications were revealed in the initial search of the four databases: Web of Science (n = 767), PubMed (n = 1310), Scopus (n = 851) and Embase (n = 664). However, 1742 duplicated records were excluded. After screening the titles and abstracts, 1650 publications were excluded because they were unrelated to the use of peptides in caries management.

The researchers retrieved and screened the full text of the 200 relevant publications. They also screened the reference lists of these publications. An additional 17 publications that potentially met the inclusion criteria were added for full-text screening. After the full-text screening of the 217 publications, 107 were excluded because they were not related to the design of novel peptides for caries management and 48 were excluded because they were not the first publication on the reported peptide. Thus, a total of 62 publications were included in our analysis.

### 2.1. Antimicrobial Peptides

The studies relating to antimicrobial peptides included 47 studies that reported 57 antimicrobial peptides. These studies used six methods to design antimicrobial peptides: the template-based design method, the conjugation method, the synthetic combinatorial technology method, the de novo design method and cyclisation. Table 1 summarises the design methods and the source of antimicrobial peptides for caries management. 

Among them, 31 studies (66%, 31/47) used the template-based design method, 9 studies (19%, 9/47) used the conjugation method, and 7 studies (15%, 7/47) used other methods. 

The source of the peptides designed using the template-based design and the conjugation methods varied. Ten studies used human-sourced peptides to develop novel peptides. Twelve studies used bacterial-sourced peptides to create novel peptides. The remaining studies used peptides from animals, such as frogs and fish, and plants, such as rice and *Punica granatum*. Table 2 shows the functional parts of peptides that were designed using the conjugation method.

Using the above-mentioned methods, these studies developed novel antimicrobial peptides for use in caries management. The functions of these antimicrobial peptides included antimicrobial activity against *S. mutans* and antibiofilm activity against *S. mutans* biofilm (Table 3).

However, the mechanisms of these peptides against *S. mutans* were different, as follows: SspB(390–T400K–402) and Ssp(A4K-A11K) competitively prevented the adhesion of *S. mutans* to the salivary pellicle on hydroxyapatite [52,64]; some peptides—such as C16G2, 2_1G2 and C11H—selectively targeted *S. mutans* [33,73,75]; other peptides—such as HBAMP, SHABP, MHABP and DPS-PI—exhibited hydroxyapatite affinity [77,78,79] and could adhere to the hydroxyapatite surface and exhibit prolonged activity against cariogenic biofilm; and some peptides showed activity against *C. albicans* [49,50,60,61,86,88].

Referring to our risk of bias assessment, most of the studies (68%, 32/47) had a medium risk of bias, 14 studies (30%) had a high risk of bias and only 1 study presented a low risk of bias.

Table 4 shows the risk of bias in the studies on antimicrobial peptides in caries management.

### 2.2. Mineralising Peptides

Ten studies reported on mineralising peptides (Table 5). Of these studies, seven (70%, 7/10) used the template-based design method; one (10%, 1/10) used the conjugation method; and two (20%, 2/10) used the de novo design method. All peptides, except the de novo designed peptides, were sourced from human beings. A novel oligopeptide was derived from the dentine matrix protein 1 and amelogenin [87]; QP5 and shADP5 were also derived from amelogenin [35,92]; StN21 and DE-11 were derived from statherin [93,94]; and 8DSS and 3NSS were derived from dentin phosphoprotein [95,96]. In addition, Cpne7-DP was derived from Cpione-7, which is a calcium-dependent phospholipid-binding protein [97].

The functions of these mineralising peptides promoted remineralisation and reduced demineralisation (Table 6). Studies on novel oligopeptides and Cpne7-DP have used dentine tissue to assess the mineralising properties of the peptides [87,97]. A study on StN21 used hydroxyapatite blocks to evaluate the mineralising properties of the peptide [93]. Other studies used enamel tissue.

Among these studies, nine (90%, 9/10) had a medium risk of the bias, one presented a high risk of bias and no studies showed a low risk of bias (Table 7).

### 2.3. Peptides with Antimicrobial and Mineralising Properties

Five studies developed their peptides to have antimicrobial and mineralising properties. They all used the conjugation method (Table 2). The functions of the peptides that had antimicrobial and mineralising properties in caries management are shown in Table 8. Two domains of DR9-PP14 were derived from histatin-3 and statherin, respectively [88]; CS-QP5 combined with QP5 and was derived from amelogenin and an antimicrobial chitosan hydrogel [89]; TVH19 consisted of a de novo designed antimicrobial peptide, GH12, and a mineralising domain that was sourced from amelogenin [90]; and *Sp*-H5 combined with phosphoserine and histatin-5 [91].

GA-KR12 was designed by grafting gallic acid—to act as a mineralising domain—to an antimicrobial peptide—KR12, which was derived from human LL-37 [38]. All peptides showed activity against *S. mutans.* DR9-RR14 and GA-KR12 also showed activity against *C. albicans* [38,88]. These studies used enamel tissues to evaluate the mineralising properties of their novel peptides.

Among them, two studies showed a low risk of bias, whereas the others had a medium risk of bias (Table 9).

### 2.4. Risk of Bias in Individual Studies

Most of these studies (44/62) showed a medium risk of bias. All the studies had negative or positive controls. In addition, most of the studies (59/62) used a standard synthetic technique to create peptides, and all the studies reported the applied method of peptides. However, very few studies (2/62) reported their sample size calculation. None of the included studies had a blind design. Approximately half of the studies reported their peptides’ characteristics (33/62) and biocompatibility (32/62). Many of the included studies (52/62) did not assess the stability of peptides in human saliva. Last, one-third of the studies (22/62) investigated the mechanisms of the peptides’ functions.

## 3. Discussion

### 3.1. Design Methods of Novel Peptides Used in Caries Management

In this study, the investigators found that the researchers in the reviewed studies used the template-based design method, the conjugation method, the synthetic combinatorial technology method, the de novo design method and cyclisation to design novel peptides for use in caries management.

#### 3.1.1. Template-Based Design Method

In this study, the investigators found that the template-based design method was the most frequently used method in developing novel peptides for use in caries management. Sequence templates can be obtained by comparing a large number of natural peptides and proteins from different sources. Several databases, such as the Collection of Anti-microbial Peptides and the Antimicrobial Peptide Database, supplied the researchers with information on the sequences of various peptides, their activities, source organisms, target organisms and applications [11]. Researchers have also specifically developed a dataset on antimicrobial peptides for use in caries management [100]. These databases are all essential tools for discovering and designing novel peptides. For example, natural peptides—such as gaegurin from frogs, kappa-casein from bovines, cecropin from insects, nisin from bacteria and statherin from humans—have been used as templates to develop novel peptides. The extracting patterns of templates are based on functional requirements. Some studies have used full-length peptides to develop their novel peptides. For example, Tong et al. used nisin from *Lactococcus lactis* as an antimicrobial peptide against *S. mutans* [53]. However, other studies have modified template peptides to develop novel peptides. The properties of the peptides—such as their secondary structure, cationic, hydrophobicity, activity, stability and biocompatibility—can be systematically changed based on the modification. D1-23 and hBD3-C15 are truncated fragments of human defensin [61,62]. In the studies that we reviewed, they both showed activity against *S. mutans*. MUC7-12mer, which shows activity against *S. mutans* and *C. albicans,* is a truncated fragment of human mucin [48].

However, further modifications were needed in the studies because the peptide fragment properties of the truncated template were not ideal. For example, SspB(390–T400K–402) was designed based on the SspB cell surface adhesin of *Streptococcus gordonii.* Furthermore, threonine (T), at position 400, was substituted with lysine (K) to increase the binding activity of the peptide. This modification gave SspB(390–T400K–402) an increased binding activity to the salivary pellicle, enabling it to inhibit the formation of *S. mutans* biofilms on a tooth’s surface [52]. QP5, derived from human amelogenin, was designed based on the mineralising sequences of Q-P-X in amelogenin and a hydrophilic tail. The function of the amelogenin was to control the orientation of enamel-rod growth during mineralisation [101]. This novel peptide was water-soluble and had calcium affinity, which promoted the remineralisation of enamel caries [35]. The template-based design method is a common method for designing novel peptides. In addition, a peptide sequence that is developed based on the template-based design method can also be used as the functional domain in the conjugation method. The abundant resources of natural peptides have inspired researchers to create novel peptides for use in caries management. However, everything has two sides. Deriving new template peptides from natural peptides can distract the focus of researchers from promoting the translation of already developed peptides to clinical applications.

#### 3.1.2. Conjugation Method

Using the conjugation method to develop functional peptides has recently been a popular research topic [15,28]. For caries management, researchers use this method to design and develop specifically targeted antimicrobial peptides against *S. mutans*, hydroxyapatite affinity peptides, and dual-action peptides with antimicrobial and mineralising properties [15]. The conjugation of differently designed or separately sourced peptides can be performed. Wang et al. grafted a de novo designed antimicrobial peptide, GH12, to a template-based mineralising peptide, TD7, to develop a novel peptide with antimicrobial and mineralising properties [90]. Eckert et al. used the bacterial-sourced peptide C16 and the sheep-sourced peptide G2 to create a novel, specifically targeted, antimicrobial peptide, which could act against *S*. *mutans* [33]. In addition, other molecules besides peptides can also be used as a domain in a conjunction peptide. For example, Niu et al. designed their peptide using gallica acid as a mineralising domain [38].

#### 3.1.3. Synthetic Combinatorial Technology Method

Synthetic combinatorial technology is the development of a combinatorial library, which consists of amino acid sequences that can be used to screen peptides for specific functions, such as their activity against *S. mutans* [80].

KSL was identified from a decapeptide library because of its ability to inhibit oral bacteria growth, such as *S. mutans,* and its biocompatibility with human gingival epithelial cells [80]. Pac-525 was identified from a tryptophan-rich peptides library due to its antimicrobial activity against *Bacillus cereus*, *Escherichia coli*, *Staphylococcus aureus* and *Pseudomonas aeruginosa* [81].

In addition, researchers designed D-Nal-Pac-525, which was based on Pac-525, by replacing all tryptophan residues with D-β-naphthylalanine. D-Nal-Pac-525 exhibited improved antimicrobial activity compared to Pac-525 by showing activity against *S. mutans* [81]. C10-KKWW was identified as being active against *S. mutans* from a lipopeptide library [82]. The difference here was that it was identified by virtual screening. The synthetic combinatorial technology method originated from the use of solid supports for peptide production [102]. The term ‘combinatorial’ comes from the size of the library, which increases with the number of reagents. The major disadvantage of this method is the high cost of its production.

#### 3.1.4. De Novo Design Method

The key component of the de novo design method is the design of specific structural peptides, which are based on the properties of amino acids. The de novo design could start from the sequence (XXYY)n, where X refers to hydrophobic amino acids and Y to cationic amino acids. The newly designed peptide has an α-helical structure. Tu et al. used GLLW+ (HLLH)n to create novel peptides [83]. The novel peptide starts with glycine (G) in the first position because it commonly acts as an N-terminus residue for α-helical natural antimicrobial peptides. Tryptophan (W) helps peptides anchor to the lipid bilayer surface. Histidine (H) is a cationic amino acid, and leucine (L) is a hydrophobic amino acid. Finally, they found that GLLW+ (HLLH)_2_ was promising and named it GH12. GH12 showed rapid and strong antimicrobial activity against *S. mutans* [83].

The de novo design could start from the sequences (yyxzxWxzxyy), as a short α-helical template (y refers to hydrophobic amino acids, x to cationic amino acids and z to other amino acids). Chou et al. designed a novel peptide, P19, to act against *C. albicans* based on this sequence. In addition, Kirkham et al. and Li et al. used alternating polar and nonpolar amino acids to design the β-sheet peptides P_11-4_ and ID8, respectively [98,99]. These peptides showed a strong tendency to self-assemble into fibrillar networks, which could form scaffold-like structures. This scaffold-like structure can induce hydroxyapatite regeneration and promote the remineralisation of initial caries. The de novo method is based on elementary physical models. Although it is not easy to develop peptides with complex functional molecules through the de novo design method, it significantly contributes to researchers’ understanding of membrane–protein folding [103].

#### 3.1.5. Cyclisation

Generally, four modes of cyclisation have been found in natural antimicrobial peptides. They are as follows: cyclisation via head-to-tail cyclisation, cyclic dipeptides, disulphide bonds and internal bonding between side chains [104,105]. Cyclisation reduces the peptides’ nonspecific cytotoxicity against human cells and improves their stability [106]. However, this method for designing novel peptides for caries management is not common. CLP-4, which shows activity against *S. mutans,* is a cyclic lipopeptide with a six-amino-acid macrocyclic ring [85]. Simon et al. identified five novel cyclic dipeptides that inhibited the growth of *C. albicans* and *S. mutans* [86].

### 3.2. Quality of Studies on Peptides for Caries Management

There is no consensus among researchers on how to effectively assess the quality of in vitro studies [107]. The methods used in our review to assess the risk of bias were adapted from a systematic review of in vitro studies [11]. High-quality in vitro studies are essential for the further translation of the developed peptides into clinical use [40,41]. This review evaluated the quality of the selected studies through an assessment of bias risk. Most of the included studies showed a medium risk of bias. Thus, it is necessary to further improve the quality of studies on novel peptides for use in caries management. The major strengths of the reviewed studies’ designs include the following: all of the studies had negative or positive controls, most of the studies used a standard synthetic technique to create the peptides and all of the studies reported the peptides’ methods of application. All of these are beneficial for guaranteeing the repeatability and reproducibility of studies. However, the limitations of these studies are also obvious to us. For example, only a few studies reported their sample size calculation and none of them set a blind design. It should be noted that a sample size calculation is essential for both in vivo and in vitro studies [108]. However, reports have also indicated that non-blinded studies tend to report more significant *p*-values [11].

In the assessment of novel peptides, a secondary structure is critical for their ability to function. Thus, confirming the secondary structure of a novel peptide after its creation is necessary. Many of the reviewed studies used circular dichroism spectroscopy [38] to analyse the secondary structure of the peptides. Although, some studies also used nuclear magnetic resonance spectroscopy [76] or computer simulation [91] to analyse the second structure. In addition, in terms of clinical application, assessing the ideal biocompatibility of the peptides is also essential. Haemolytic activity is a major side effect of peptides [109]; however, because the novel peptides for use in caries management will be applied in the oral cavity, haemolytic activity is not a concern. Erythrocytes and human gingival fibroblasts were the most used cell lines in the studies’ biocompatibility testing. However, some studies still used non-human cells [84] or non-oral cells [79]. Furthermore, the main limitation of all these studies in terms of their assessment of novel peptides was that many of them did not assess the stability of the peptides in human saliva. This is important because natural peptides are generally unstable in saliva [22], and one of the primary purposes of introducing novel peptides is to increase their stability [38]. Thus, assessing their stability is essential for studies on novel peptides for use in caries management. In addition, not all the studies investigated the mechanisms of the peptides’ functions. However, it should be noted that one of the inclusion criteria for this systematic review was that only the first study on a specific peptide should be used; therefore, not all of the studies will have performed deep research on the mechanisms of the peptides’ functions at such an early stage. Overall, different methods should be used in future studies to develop novel peptides for use in caries management. However, a standard test process for these novel peptides is necessary.

## 4. Materials and Methods

### 4.1. Eligibility Criteria

The eligibility criteria for this systematic review were as follows:

- Original in vitro studies;
- Studies focused on developing novel peptides for the management of caries;
- The first study on a specific novel peptide for managing caries.

It should be noted that this study focused on the different designs of peptides. Therefore, this systematic review only included the first study on each specific peptide.

### 4.2. Exclusion Criteria

- Literature reviews;
- Conference abstracts;
- Clinical case reports;
- Case series;
- Studies unrelated to caries or peptides;
- Studies without full papers;
- Not being the first study on a specific novel peptide for managing caries.

### 4.3. Search Strategies

**Figure 1:**
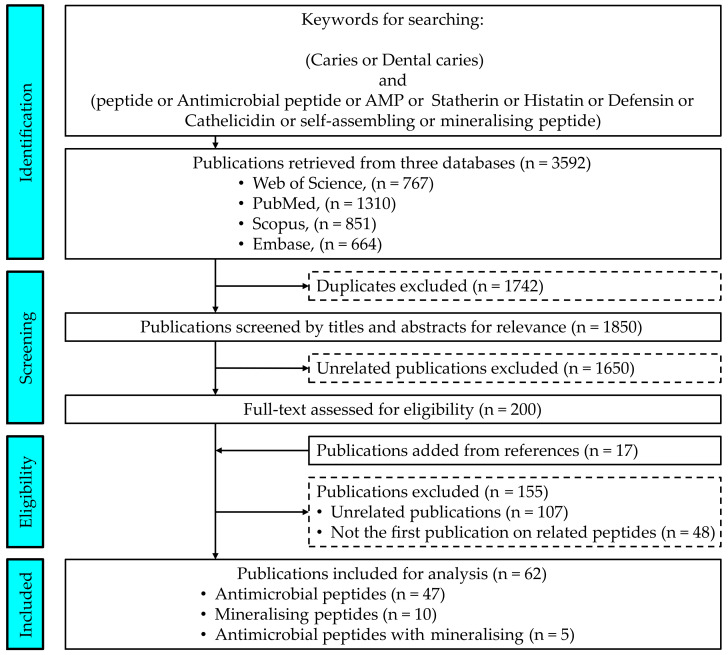
Flowchart of the study.

The problem/population, intervention, comparison and outcome (PICO) elements [110] are important to appropriately choose an information resource and craft a search strategy for systematic review. However, this study did not focus on a clinical problem and we could not use PICO as the framework to develop a question. Furthermore, I, C and O did not fit well. Therefore, we adapted PICO accordingly as, “In studies performed in vitro (P), are the methods (C) for the creation of peptides for caries management (I) differently designed (O)?” The keywords in the search were as follows: ‘“caries” OR “dental caries”’ AND ‘“peptide” OR “antimicrobial peptide” OR “AMP” OR “Statherin” OR “Histatin” OR “Defensin” OR “Cathelicidin” OR “self-assembling” OR “mineralising peptide”’. Two investigators independently searched through the literature to identify English publications, using four common databases: Web of Science, Pubmed, Scopus and Embase (Figure 1). They performed their last search on 30 November 2022.

### 4.4. Study Selection and Data Extraction

The investigators removed the duplicated records. They then screened the titles and abstracts to choose publications that were potentially eligible. Literature reviews, conference abstracts, clinical case reports, case series, studies unrelated to caries or peptides and studies without full papers were excluded. Later, full texts were rigorously selected for articles that satisfied the inclusion criteria. All reference lists were examined through manual screening to seek publications that potentially met the inclusion criteria. When a series of studies on the same peptide were found, we only included the first publication on the design of the peptide in question. Disagreements on including or excluding publications were resolved through discussions with a third investigator. Data were extracted from all included publications. The information collected included authors and years, the names of peptides, design methods for peptides, the source of peptides, the function of peptides in caries management and other main findings.

### 4.5. Assessment of the Risk of Bias in Individual Studies

Two independent investigators assessed the risk of bias in each study. The assessment items were adapted from previous systematic reviews [11]. The following nine items were considered in all of the publications that we reviewed:

- Item 1: presence of control;
- Item 2: description of sample size calculation;
- Item 3: peptide synthesis using standard methods;
- Item 4: characterisation of peptides;
- Item 5: assessment of peptide stability;
- Item 6: assessment of peptide biocompatibility;
- Item 7: application methods of peptides;
- Item 8: investigation of peptide mechanisms;
- Item 9: blinding of observers.

Publications that reported fewer than four of these items were classified as having a high risk of bias, whereas those reporting more than six were classified as low risk. Disagreements were resolved through discussion.

## 5. Conclusions

Researchers have used different methods to develop peptides for use in caries management. The most common methods were found to be the template-based design method and the conjugation method. In the future, researchers should use these methods to generate more novel peptides for use in caries management. In addition, the quality of any future studies on using peptides for caries management should improve on those that have already been conducted.

## Figures and Tables

**Table 1:** Design methods and the source of antimicrobial peptides for caries management.

Design Methods and the Source of Peptides	Authors, Year [Reference Number]
***Template-based design method***	
GGN6, frog: gaegurin	Kim et al., 2003 [42]
Kappacin, bovine: kappa-casein	Dashper et al., 2005 [43]
Cecropin-XY, insect: cecropin	Hao et al., 2005 [44]
K4 -S4(1-15)a, tree frog: dermaseptin	Altman et al,. 2006 [45]
PsVP-10, *Pseudomonas* sp.: R10	Padilla et al., 2006 [46]
dhvar5, human beings: histatin	Szynol et al., 2006 [47]
MUC7-12mer, human beings: mucin	Wei et al., 2006 [48]
mPE, frog: magainin	Beckloff et al., 2007 [49]
CSA-13, human beings: ceragenin	Isogai et al., 2009 [50]
AAP, styela clava: clavanin A	Li et al., 2010 [51]
SspB(390–T400K–402), *S gordonii*: Ssp	Okuda et al., 2010 [52]
Nisin, bacteria: nisin	Tong et al., 2010 [53]
hLF1–11, human beings: lactoferrin	Huo et al., 2011 [54]
Pleurocidin, fish: pleurocidin	Tao et al., 2011 [55]
chrysophsin-1, fish: chrysophsin	Wang et al., 2012 [56]
Lys-a1, frog	da Silva et al., 2013 [57]
Bac8c, bovine: bactenecin	Ding et al., 2014 [58]
L-K6, frog: temporin-1	Shang et al., 2014 [59]
Amyl-1–18, rice: α-amylase	Taniguchi et al., 2015 [60]
D1–23, human beings: defensin	Kreling et al., 2016 [61]
hBD3-C15, human beings: defensin	Ahn et al., 2017 [62]
ZXR-2, insect: mauriporin	Chen et al., 2017 [63]
KR12-KAKE, human beings: cathelicidin	da Silva et al., 2017 [31]
Ssp(A4K-A11K), *S gordonii*: Ssp	Ito et al., 2017 [64]
IG-13-1 and IG-13-2, human beings: cathelicidin	Chen et al., 2019 [65]
LR-10, *Lactobacillus* sp.: reutericin 6	Liang et al., 2019 [66]
Pug-1, *Punica granatum*	Kokilakanit et al., 2020 [67]
GHaR6R, GHaR7R, GHaR8R, and GHaR9W, frog: temporin	Wei et al., 2020 [68]
Gj-CATH2, gekko: cathelicidin	Cai et al., 2021 [69]
LF-1 and LF-2, human beings: lactoferrin	Luo et al., 2021 [70]
LFA-LFC, camel milk	Mohammadipour et al., 2021 [71]
***Conjugation method***	
C16G2, *details in* Table 2	Eckert et al., 2006 [33]
M8(KH)-20, *details in* Table 2	He et al., 2009 [72]
2_1G2, *details in* Table 2	Li et al., 2010 [73]
Sm6(L1)B33, *details in* Table 2	He et al., 2010 [74]
C11H, *details in* Table 2	Huo et al., 2018 [75]
IMB-2, *details in* Table 2	Mai et al., 2011 [76]
HBAMP, *details in* Table 2	Huang et al., 2016 [77]
SHABP and MHABP, *details in* Table 2	Yang et al., 2019 [78]
DPS-PI, *details in* Table 2	Zhang et al., 2019 [79]
***Synthetic combinatorial technology method***	
KSL, decapeptide library	Concannon et al., 2003 [80]
D-Nal-Pac-525, tryptophan-rich peptides library	Li et al., 2013 [81]
C10-KKWW, lipopeptide library	Xiang et al., 2019 [82]
***De novo design method***	
GH12	Tu et al., 2016 [83]
P19	Chou et al., 2021 [84]
***Cyclisation*** ***method***	
CLP-4, bacterial: fusaricidin	Min et al., 2017 [85]
39a, 39b, 39c, 41a, and 41b, cyclic dipeptides	Simon et al., 2019 [86]

**Table 2:** Functional parts of conjugation method-designed peptides for caries management.

Peptides [Ref. No.]	Function Domain I and Source	Function Domain II and Source
C16G2 [33]	*S. mutans* targeting domain; *S. mutans*: competence-stimulating peptide	Antimicrobial domain; sheep: antimicrobial peptide 29
M8(KH)-20 [72]	*S. mutans* targeting domain; *S. mutans*: competence-stimulating peptide	*Pseudomonas* spp. targeting domain; KH peptide
2_1G2 [73]	*S. mutans* targeting domain; 2_1	Antimicrobial domain; sheep: antimicrobial peptide 29
Sm6(L1)B33 [74]	*S. mutans* targeting domain; Sm6	Antimicrobial domain; peptides binary libraries
C11H [75]	*S. mutans* targeting domain; *S. mutans*: competence-stimulating peptide	Antimicrobial domain; human beings: lactoferrin
IMB-2 [76]	*S. mutans* targeting domain; *S. mutans*: competence-stimulating peptide	Antimicrobial domain; marine flatfish: pleurocidin
HBAMP [77]	Hydroxyapatite-binding domain; HBP7	Antimicrobial domain; KSLW
SHABP [78]	Hydroxyapatite-binding domain; CNPGFAQAC	Antimicrobial domain; 1018
MHABP [78]	Hydroxyapatite-binding domain; CMLPHHGAC	Antimicrobial domain; 1018
DPS-PI [79]	Hydroxyapatite-binding domain; phosphoserine	Antimicrobial domain; horseshoe crab: polyphemusin I
Novel oligopeptide [87]	Mineralising domain; human beings: dentine matrix protein 1	Mineralising domain; human beings: amelogenin
DR9-RR14 [88]	Mineralising domain; human beings: statherin	Antimicrobial domain; human beings: histatin-3
CS-QP5 [89]	Mineralising domain; human beings: amelogenin	Antimicrobial domain; antimicrobial chitosan hydrogel
TVH19 [90]	Mineralising domain; human beings: amelogenin	Antimicrobial domain; GH12
Sp−H5 [91]	Mineralising domain; phosphoserine	Antimicrobial domain; human beings: histatin-5
GA-KR12 [38]	Mineralising domain; gallic acid	Antimicrobial domain; human beings: LL-37

**Table 3:** Functions of antimicrobial peptides for caries management.

Functions of Peptiedes [Reference Number]		
***Inhibition of S. mutans growth in the planktonic phase***		
Amyl-1–18 [60]	AAP [51]	CSA-13 [50]
dhvar5 [47]	GGN6 [42]	LF-1 and LF-2 [70]
LFA-LFC [71]	M8(KH)-20 [72]	Nisin [53]
PsVP-10 [46]	Pug-1 [67]	
***Inhibition of S. mutans growth in the planktonic phase and biofilm***		
2_1G2 [73]	39a, 39b, 39c, 41a, 41b [86]	Bac8c [58]
C10-KKWW [82]	C11H [75]	C16G2 [33]
Cecropin-XY [44]	chrysophsin-1 [56]	CLP-4 [85]
D-Nal-Pac-525 [81]	D1–23 [61]	DPS-PI [79]
GH12 [83]	GHaR6R, GHaR7R, GHaR8R, and GHaR9W [68]	
Gj-CATH2 [69]	hBD3-C15 [62]	hLF1–11 [54]
HBAMP [77]	IG-13-1 and IG-13-2 [65]	IMB-2 [76]
K4 -S4(1-15)a [45]	Kappacin [43]	KR12-KAKE [31]
KSL [80]	L-K6 [59]	LR-10 [66]
Lys-a1 [57]	mPE [49]	MUC7-12mer [48]
Pleurocidin [55]	Sm6(L1)B33 [74]	ZXR-2 [63]
***Killing of S. mutans in multispecies biofilm***		
2_1G2 [73]	C11H [75]	C16G2 [33]
***Inhibition of S. mutans biofilm formation on hydroxyapatite***		
SHABP and MHABP [78]	Ssp(A4K-A11K) [64]	SspB(390–T400K–402) [52]
***Inhibition of C. albicans growth in the planktonic phase***		
Amyl-1–18 [60]	L-K6 [59]	mPE [49]
MUC7-12mer [48]	P19 [84]	
***Inhibition of C. albicans growth in the planktonic phase and biofilm***		
39a, 39b, 39c, 41a, 41b [86]		

**Table 4:** Risk of bias in the studies of antimicrobial peptides in caries management.

No.	Study Authors, Year [Reference Number]	Item #									Score	Risk of Bias
		1	2	3	4	5	6	7	8	9		
1	Chou et al., 2021 [84]	●		●	●	●	●	●	●		7	Low
2	Luo et al., 2021 [70]	●	●	●	●		●	●			6	Medium
3	Cai et al., 2021 [69]	●		●		●	●	●	●		6	Medium
4	Wei et al., 2020 [68]	●		●	●		●	●	●		6	Medium
5	Chen et al., 2019 [65]	●		●	●		●	●	●		6	Medium
6	Chen et al., 2017 [63]	●		●	●		●	●	●		6	Medium
7	Min et al., 2017 [85]	●		●	●	●	●	●			6	Medium
8	Huang et al., 2016 [77]	●		●	●	●	●	●			6	Medium
9	Shang et al., 2014 [59]	●		●	●		●	●	●		6	Medium
10	Li et al., 2010 [51]	●		●	●	●	●	●			6	Medium
11	Dashper et al., 2005 [43]	●		●	●		●	●	●		6	Medium
12	Kokilakanit et al., 2020 [67]	●		●			●	●	●		5	Medium
13	Liang et al., 2019 [66]	●		●			●	●	●		5	Medium
14	Yang et al., 2019 [78]	●		●	●			●	●		5	Medium
15	Zhang et al., 2019 [79]	●		●	●		●	●			5	Medium
16	Huo et al., 2018 [75]	●		●	●			●	●		5	Medium
17	Xiang et al., 2019 [82]	●		●	●			●	●		5	Medium
18	da Silva et al., 2017 [31]	●		●	●			●	●		5	Medium
19	Ding et al., 2014 [58]	●		●			●	●	●		5	Medium
20	Li et al., 2013 [81]	●		●	●			●	●		5	Medium
21	Wang et al., 2012 [56]	●		●			●	●	●		5	Medium
22	Mai et al., 2011 [76]	●		●	●		●	●			5	Medium
23	Hao et al., 2005 [44]	●		●			●	●	●		5	Medium
24	Concannon et al., 2003 [80]	●		●	●		●	●			5	Medium
25	Kim et al., 2003 [42]	●		●	●		●	●			5	Medium
26	Mohammadipour et al., 2021 [71]	●		●			●	●			4	Medium
27	Kreling et al., 2016 [61]	●		●			●	●			4	Medium
28	Taniguchi et al., 2015 [60]	●		●			●	●			4	Medium
29	Tao et al., 2011 [55]	●		●		●		●			4	Medium
30	Huo et al., 2011 [54]	●		●			●	●			4	Medium
31	Tong et al., 2010 [53]	●		●				●	●		4	Medium
32	Wei et al., 2006 [48]	●		●	●			●			4	Medium
33	Eckert et al., 2006 [33]	●		●	●			●			4	Medium
34	Simon et al., 2019 [86]	●		●				●			3	High
35	Ahn et al., 2017 [62]	●		●				●			3	High
36	Ito et al., 2017 [64]	●		●				●			3	High
37	Tu et al., 2016 [83]	●				●		●			3	High
38	da Silva et al., 2013 [57]	●		●				●			3	High
39	Okuda et al., 2010 [52]	●		●				●			3	High
40	Li et al., 2010 [73]	●		●				●			3	High
41	He et al., 2010 [74]	●		●				●			3	High
42	He et al., 2009 [72]	●		●				●			3	High
43	Isogai et al., 2009 [50]	●		●				●			3	High
44	Beckloff et al., 2007 [49]	●						●	●		3	High
45	Szynol et al., 2006 [47]	●		●				●			3	High
46	Altman et al., 2006 [45]	●		●							2	High
47	Padilla et al., 2006 [46]	●						●			2	High

# Item 1—presence of control; Item 2—description of sample size calculation; Item 3—peptide synthesis using standard methods; Item 4—characterisation of peptides; Item 5—assessment of peptide stability; Item 6—assessment of peptide biocompatibility; Item 7—application methods of peptides; Item 8—investigation of peptide mechanisms; and Item 9—blinding of observers.

**Table 5:** Design methods and the source of mineralising peptides for caries management.

Design Methods and the Source of Peptides	Authors, Year [Reference Number]
***Template-based design method***	
StN21, human beings: statherin	Kosoric et al., 2007 [93]
8DSS, human beings: dentin phosphoprotein	Hsu et al., 2011 [96]
3NSS, human beings: dentin phosphoprotein	Chung et al., 2013 [95]
QP5, human beings: amelogenin	Lv et al., 2015 [35]
shADP5, human beings: amelogenin	Dogan et al., 2018 [92]
DE-11, human beings: statherin	Wang et al., 2018 [94]
Cpne7-DP, human beings: Copine 7	Lee et al., 2020 [97]
***Conjugation method***	
Novel oligopeptide, *details in* Table 2	Cao et al., 2014 [87]
**De novo design method**	
P_11-4_	Kirkham et al., 2007 [98]
ID8	Li et al., 2020 [99]

**Table 6:** Functions of mineralising peptides in caries management.

Functions of Peptides [Reference Number]		
***Reduction of mineral loss in hydroxyapatite***		
StN21 [93]		
***Reconstruction of the interrod regions in enamel caries***		
3NSS [95]		
***Promotion of enamel caries remineralisation***		
8DSS [96]	DE-11 [94]	ID8 [99]
P_11-4_ [98]	QP5 [35]	shADP5 [92]
***Promotion of dentine caries remineralisation***		
Cpne7-DP [97]	novel oligopeptide [87]	

**Table 7:** Risk of bias in studies on mineralising peptides in caries management.

No.	Study Authors, Year [Reference Number]	Item #									Score	Risk of Bias
		1	2	3	4	5	6	7	8	9		
1	Li et al., 2020 [99]	●		●	●		●	●			5	Medium
2	Lee et al., 2020 [97]	●		●			●	●	●		5	Medium
3	Wang et al., 2018 [94]	●		●	●		●	●			5	Medium
4	Dogan et al., 2018 [92]	●		●	●			●			4	Medium
5	Lv et al., 2015 [35]	●		●	●			●			4	Medium
6	Cao et al., 2014 [87]	●		●	●			●			4	Medium
7	Hsu et al., 2011 [96]	●		●			●	●			4	Medium
8	Kirkham et al., 2007 [98]	●		●	●			●			4	Medium
9	Kosoric et al., 2007 [93]	●		●	●			●			4	Medium
10	Chung et al., 2013 [95]	●		●				●			3	High

# Item 1—presence of control; Item 2—description of sample size calculation; Item 3—peptide synthesis using standard methods; Item 4—characterisation of peptides; Item 5—assessment of peptide stability; Item 6—assessment of peptide biocompatibility; Item 7—application methods of peptides; Item 8—investigation of peptide mechanisms; and Item 9—blinding of observers.

**Table 8:** Functions of peptides with antimicrobial and mineralising properties for caries management.

Peptides (Authors, Year) [Reference Number]	Functions for Caries Management
DR9-RR14 (Basiri et al., 2017) [88]	Inhibition of *S. mutans* growth in planktonic phase Inhibition of *C. albicans* growth in planktonic phase Prevention of enamel demineralisation
TVH19 (Wang et al., 2019) [90]	Inhibition of *S. mutans* growth in planktonic phase and biofilm Promotion of enamel caries remineralisation
Sp−H5 (Zhou et al., 2020) [91]	Inhibition of *S. mutans* growth in planktonic phase and biofilm Promotion of enamel caries remineralisation
GA-KR12 (Niu et al., 2021) [38]	Inhibition of *S. mutans* growth in planktonic phase Inhibition of *C. albicans* growth in planktonic phase Promotion of enamel caries remineralisation
CS-QP5 (Ren et al., 2019) [89]	Inhibition of *S. mutans* growth in planktonic phase and biofilm Promotion of enamel caries remineralisation

**Table 9:** Risk of bias in the studies on peptides with antimicrobial and mineralising properties in caries management.

No.	Study Authors, Year [Reference Number]	Item #									Score	Risk of Bias
		1	2	3	4	5	6	7	8	9		
1	Niu et al., 2021 [38]	●	●	●	●	●	●	●	●		8	Low
2	Zhou et al., 2020 [91]	●		●	●	●	●	●	●		7	Low
3	Wang et al.,2019 [90]	●		●	●	●	●	●			6	Medium
4	Ren et al., 2019 [89]	●		●	●		●	●			5	Medium
5	Basiri et al., 2017 [88]	●		●	●			●			4	Medium

# Item 1—presence of control; Item 2—description of sample size calculation; Item 3—peptide synthesis using standard methods; Item 4—characterisation of peptides; Item 5—assessment of peptide stability; Item 6—assessment of peptide biocompatibility; Item 7—application methods of peptides; Item 8—investigation of peptide mechanisms; and Item 9—blinding of observers.

## Data Availability

Data is contained within the article.
